# The dynamic effect of macroeconomic factors on housing prices: Evidence from South Africa

**DOI:** 10.1371/journal.pone.0290552

**Published:** 2023-11-29

**Authors:** Thami Innocent Lekhuleni, Godfrey Ndlovu

**Affiliations:** School of Economics, University of Cape Town, Cape Town, South Africa; Universidad de Almeria, SPAIN

## Abstract

This study examines the dynamic short- and long-run causal relationship between South African real house prices and key macroeconomic fundamentals (gross domestic product(GDP), mortgage rate, exchange rate-USDZAR, affordability, household debt to disposable income, unemployment rate, share prices (JSE ALL share index), foreign direct investment, and producer price index) over the period 2000Q1-2019Q4. The study uses a vector error correction model (VECM) to estimate the relationships while accounting for endogeneity and reverse causality. Although, there seems to be a significant association(both short and long-run) between house prices and all macroeconomic fundamental variables, GDP and producer price index appear to have the greatest impact. Further, our results suggest that any short-term disequilibrium in house prices always self-corrects in the long-run.

## 1. Introduction

The South African residential housing market price growth has displayed patterns of widespread price fluctuations in recent years. According to the global property guide data, South African real house prices increased by an average of 20 percent annually, from 2000 to 2006, reaching a peak of 32.5 percent in October 2004 [1] However, during the 2007–2020 period, South African housing market prices rose by approximately 62%, and when adjusted for inflation, real prices fell by 18% and the gap between nominal and real prices narrowed between 2019 and 2021 raising the question of whether South African house prices reflect fundaments [1] It is therefore the contribution of this study to try and capture and explain some of the complexities of the drivers behind South African housing market prices and how house prices adjust to the long-run equilibrium prices. This study also uses a more extended time series data than previous studies on the South African housing market.

Theoretically, house prices are determined by demand and supply factors [2] This would suggest that all other factors constant, house price movement should be explained by the relevant economic fundamentals. Therefore, it is important to understand the long-run equilibrium between house prices and macroeconomic fundamentals [3] Further, any adverse deviations from the equilibrium price levels may result in housing bubbles, with severe ramifications on the overall economy.

In an attempt understand housing price dynamics, two main approaches have been emerged: the microeconomic and macroeconomic approach. Microeconomic-based studies, for example, [4–6] focus on household-level factors and/or surrounding environment characteristics that affect house prices. The key assumption is that houses are heterogenous in nature and their value is determined by the buyer’s evaluation of the house’s inherent attributes such as appearance and location. However, the microeconomic approach is data consuming, requires a lot of model assumptions and often transactional data is not readily available, where available, it is costly to obtain. Further, it is often difficult to determine the model’s functional form which requires experience and judgement [7, 8]

On the other hand, the macroeconomic approach analyzes the impact of macroeconomic factors on house prices [3, 9–12] However, early research on the impact of macroeconomic factors on the housing market (for example [13–16]) did not account for the fact that the macroeconomic variables are also affected by supply and demand shocks in the housing market [17]. was amongst the first to develop a study that accounts for the dynamic interaction between the housing maker and the entire economy.

Over the past decades, there has been an increase in the number of studies on residential house prices over the past decades, as they play major role in both developed and developing. From an investment perspective, an effective understanding of the impact of the housing market on other economic variables is key as household holdings in the housing market often constitute a largest proportion of household wealth. Most of these studies suggest the existence of a non-linear long-run relationship between house prices and their fundamental determinants. They also find evidence of reverse causality and endogeneity between house prices and their determinants. The easily accessible macroeconomic data and less complexity of the models that require fewer model assumptions in the macro-economic approach compared to the microeconomic approach, makes the macro-economic approach more ideal for this study.

Although a number of studies have looked at housing price dynamics, there has been mixed evidence, which suggests that a most issues relating to house prices and economic fundamentals have not been fully explored. Further, most of these studies tend to focus on highly advanced economies and there is limited empirical evidenced based on contemporary data and the recent price movements in countries like South Africa.

This study seeks to examine the short-run and long-run causal relationship between South African house prices and selected macroeconomic fundamentals: gross domestic product (GDP), mortgage rate, exchange rate-USDZAR, affordability, household debt to disposable income, unemployment rate, share prices (JSE ALL share index), foreign direct investment (FDI), and producer price index (PPI) over the period 2000Q1-2019Q4. Given that house prices in the short run have been previously observed to deviate from their long-run equilibrium and may continually readjust through an error correction mechanism as observed from many previous studies. Therefore, a tool that is well suited to simultaneously analyse short-run dynamics as well as the long-run relationship of cointegrated variables while accounting for while accounting for reverse causality and endogeneity is needed. Therefore, this study employs a Vector error correction model to examine the co-movement between housing price and economic fundamentals and their dynamic relationship both in the long and short run. Further, we analyse the deviation of house prices from their fundamentals and thus examine the response of real house prices to macroeconomic shocks using variance decompositions and impulse response functions.

Unlike previous South African studies that focused mainly on the long-run behaviour of provincial-level house prices, their persistence and ripple effect on the economy [18,19] (, or demographic factors that affect house prices [12], this study uses the latest national-level house price data and analyzing both the long-run and short-run dynamics between house prices and macroeconomic fundamentals while accounting for reverse causality and endogeneity using the VECM. The superior capability of the VECM in modelling short-run and long-run relationship between real house prices and macroeconomic factors and thus provide more insight on policy and investment decisions on stabilizing the housing market and when to enter or exit the market.

Our results suggest existence of a long run relationship between South African real house prices and their macroeconomic fundamentals. Specifically, the gross domestic product and producer price index were found to be the most significant drivers of South African house prices compared to all other variables under review. It was also observed that any deviation of house prices from their macroeconomic fundamentals in the short-run self-corrected in the long-run. The variance decomposition results suggest that real house prices are the greatest source of forecast error variation to future real house prices. The impulse response functions suggest that a one standard deviation shock from the mortgage rate, household debt to disposable income, share prices, and foreign direct investment has a negative and persistence effect on house prices. In addition, after the shock, real house prices maintained a lower equilibrium level than the initial equilibrium level. While the rest of the variables shock left real house prices at a much higher equilibrium level.

The remainder of the paper is structured as follows. Section 2 presents a brief review of selected literature that examines the relationship between house prices and its determinants. Section 3 describes the data and the methodology. Section 4 is an analysis of empirical findings and discussion of the results. Section 5 provides a summary of conclusions and recommendations.

## 2. Literature review

### 2.1 South African housing market and macroeconomic determinants

The South African residential housing market is the largest component of its property market and accounts for a considerable amount of household wealth. According to the Centre for Affordable Housing in Africa, the demand for residential housing increased by approximately 15 per cent and 100 per cent over the 2008–2019 period, for the affordable house market (houses worth R300 000-R600 000) and the luxury market (houses worth over R1.2 million); respectively.

In addition, the house resale market in South Africa far exceeds the new built market, where in 2019 roughly three quarters of all residential transactions were in the resale market and most of the residential houses were either purchased through mortgage loans from banks or subsidised from various government subsidy programmes [20]). Considering how much wealth is invested in the South African residential housing market and how dynamic house prices can be, a great amount of literature examining the residential housing sector is expected. However, currently in South Africa, such literature is severely limited and out of those that exist only few examine the drivers of South African house prices. For instance, [21] developed a regression model for the purpose of identifying key drivers of the South African residential property market. They found that foreign direct investment positively affects house prices where a 1 percent increase in foreign direct investment was associated with a 0.12 percent increase in house price growth rate. Furthermore, their model revealed that the depreciation of the value of the rand against the U.S dollar was associated with the increase in house prices during the 1974–2003 period [22]. who’s study used a similar modelling approach to that of [21], also observed a negative relationship between the rand/dollar exchange rate and house price growth rates, where a 1% increase in the rand/dollar exchange rate was associated with a 0,04% decrease in the quarterly house price growth rate. Their analysis further revealed the existence of a positive (negative) relationship between lagged values of gross domestic product (prime rate of interest) and house prices, where a 1% increase in GDP (prime rate of interest) was associated with a 0,367% (0,026%) increase (decrease) in the quarterly house price growth rate.

[12] examined the demographic effects on South African house prices using provincial-level data set over the period from 1995 to 2015 while controlling for spatial effects, endogeneity and heterogeneity. Their analysis revealed that in the past 22 years, on average, population aging has led to the decline of South African house prices by approximately 7.52 and 6.28 basis points in medium and large housing segments, respectively, while the small segment has remained unaffected. Furthermore, unemployment appeared to have had a negative effect on the growth rate of house prices. Utilising a non-parametric approach [19], examined the short- and long-run relationship between South African real stock and house prices while controlling for structural breaks. The model showed a positive bi-directional relationship between stock and house prices. Their results were consistent with [22] who found observed a 0.034 percent increase in house prices from a 1 percent increase in lagged stock market returns.

While examining the contractionary monetary policy and its effect on house prices during the 1980Q1 to 2006Q4 period, [23]’s factor-augmented vector autoregression model (FAVA) showed an inverse relationship between house prices and monetary policy shock. This was consistent with the finding of [24] who’s structural vector autoregressive (SVAR) model revealed a persistence decline in house prices that is associated with a contractionary monetary policy. In contrast to [23–25] SVAR model results showed that house prices are exogenous and, at least, are not driven by monetary policy shocks. The deviation of [25] results from economic theory might be attributable to the limited number of variables and the type of model they used, while [23] FAVA models is able to exploit a large data set and numerous variables without losing accuracy in its prediction.

### 2.2 Macroeconomic determinants of house prices in developing and advanced economies

The boom-and-bust patterns in real estate have contributed to the fragility of the financial system in previous crises and the recent record high house prices in several property markets around the world. This has ignited much discussion in economic research and an increase in international literature that adopts a broad range of methodologies to determine key macroeconomic fundamental house price drivers to try and explain whether the current and previous price developments can be regarded as macroeconomic fundamentally justified or not.

Against this backdrop, the gross domestic product (GDP) is regarded as one of the potential macroeconomic fundamental determinants of house prices. For instance [26], applied a multivariate linear regression on Australian house prices and found that GDP has a significant and positive effect on house prices during the 1986–2004. period. Their study was further corroborated by [3] their study found a positive and statistically significant long-run relationship between house prices in Sydney and GDP [27]. examined the effect of key macroeconomic London house price drivers in the United Kingdom over the period 1971–2012. Their error-correction model revealed that fundamentals such as GDP are positively and significantly involved in estimating house prices in the United Kingdom. Similarly, [28] who used the same method as [27] found the existence of a positive long run cointegration association between GDP and house prices in the United Kingdom.

The latter mentioned studies only involve countries with advance economies, however, similar results are also observed from countries with emerging economies. A study by [29] showed GDP having a positive and strong impact on Malaysian house prices in the short-and long-run during the 2002Q1-2015Q4 period. Conversely [30], results seem to indicate that Malaysian house prices are not significantly driven by GDP. Using a Vector Auto-Regression (VAR) model framework based on the 1999Q2-2009Q3 China house price data, [31] found a strong influence of real GDP growth rate on house prices. However, a study by [32] who used a panel data regression analysis framework based on the 2000 to 2018 annual house price data, did not find any significance influence of real GDP growth rate on Chinese house prices.

Interest and mortgage rates are theoretically an important factor that shapes the demand for housing and ultimately affecting house prices. This was observed by [11], who’s vector autoregression model showed a strong and negative causal relationship between real mortgage interest rate and real house prices in Sweden. Similar estimation results were obtained by [33] in 3 major cities (Stockholm, Gothenburg, and Malmo) in Sweden where a one percent decrease in mortgage interest rate was associated with a 0.456 percent increase in house prices. Using a vector error correction model approach to examine house prices in Australia, Canada and New Zealand [34], observed a high and significant effect of the real mortgage rate on house prices across the three countries, where for instance, in Australia, a 1 percentage point increase in the interest rate is associated with a 5 to 9 percent fall in house prices. In emerging economies such as China [35], observed similar directional effects, where in the long run, the mortgage rate was associated with a significant negative effect on seven major cities in China(i.e. Beijing, Shanghai, Tianjin, Guangzhou, Shenzhen, Hangzhou and Chengdu.)

The link between the stock and house market and the pivotal role they play in a country’s economy has resulted in stock prices being a crucial factor behind the movement of house prices through the wealth effect [36]. For instance [37] applied a bootstrap panel Granger causality approach to examine the existence of long-run causal relationship between stock and house prices for seven major European countries during the 1975Q1-2017Q1 period. Their results show a positive and unidirectional causality from stock prices to house prices in France, Netherlands, Italy, UK and Sweden. In contrast, their results also showed a significant negative impact of stock prices on house prices for Germany and Switzerland. [38]’s granger causality test revealed the existence of a wealth effect in most Asian emerging markets (China, Singapore, Taiwan, and Hong Kong) where stock prices had a positive impact on house prices and a negative impact on South Korea house prices.

Many Investors regard the exchange rate as an important component in the evaluation of the housing market and any movement in exchange rates affects the property investment decision of investors which impacts house prices. This was observed by [39], who’s Autoregressive Distributive Lag (ARDL) model showed that an appreciation of the US dollar against the Turkish Lira had a significant and increasing effect on Turkish house prices during the 2010–2020 period. In contrast, [40], who used a Vector Autoregression (VAR) Model did not find any significant effect of the US dollar Turkish Lira exchange rate on Turkish house prices [41]. found that in an emerging and open economy such as India, an appreciation of the real effective exchange rate was associated with a decline in house prices. Another study from an emerging economy, was that of [42] who used a Vector Autoregression (VAR) Model found that an appreciation of the US dollar against Chinese yen exchange rate was associated with an increase in house prices.

While examining factors that drive house prices in developed economies, [43], vector error correction model (VECM) showed a positive and significant relationship between real house prices and real disposable income in the long run for the following developed economies: United Kingdom, Japan, New Zealand, Norway, Sweden, Switzerland, Canada, Belgium, Germany, Ireland, Spain, and Italy during the 1970 to 2011 period. [43]’s results were in line with those of [44] who found that real per-capita disposable income (RPDI) has a positive correlation with house prices among advanced economies (add footnote of the countries). However, studies such as those of [30], deviate from the theoretical assumption of the positive impact of disposable income on house prices. Their VECM results established that the level of gross domestic product and household disposable income do not significantly affect Malaysian house price growth.

Applying the Australian data for 1995Q4 to 2015Q3, [45] uses the VECM to investigate principal drivers of Australian house prices. Their empirical results revealed a positive but insignificant impact of the unemployment rate on house prices with a positive coefficient of 1.93. In contrast,[3] found a negative and significant relationship between Australian house prices and unemployment rate, where a one-unit increase in the unemployment rate is associated with a 0.824 decrease in median house prices. [27]’s results deviated from economic theory, where unemployment rate was found to have a positive and significant effect on UK house prices using an error correction model based on the quarterly data from 1971Q1 to 2012Q4.

In recent years, there has been a growing interest in examining the linkages between capital inflows, namely, foreign real estate investment and House prices in both developed and emerging economies. For instance, using data from 21 emerging economies, [46]’s panel VAR model found that higher foreign investment stimulates economic growth and leads to an increase in house prices over the 2000–2008 period. In a related study, [47] showed that capital inflows have a significant and positive impact on economic growth and house prices in Asian emerging market economies over the 2000–2011 period. In emerged economies, a study by [48] showed a positive and significant impact of capital inflows and expansionary monetary policy on house prices from OECD (Organisation for Economic Co-operation and Development) countries over the 1984–2006 period. However, in a similar study, [49] did not find any impact of foreign direct investment on house prices in both the short run and long run from a set of OECD countries between 1995 and 2008.

The studies reviewed above provide valuable insights in understanding key macroeconomic fundamental drivers of house prices both in emerging and emerged economies. However, their findings should be interpreted with caution. Firstly, it is observed that they use different models to reach different findings where some variables are significant in one study, but not significant in another. For instance, it is noted that most studies applied a Vector autoregression (VAR) model or a modified version of it and some of these studies do not follow the necessary steps needed to apply a VAR model such as using statistical methods to select the best lag order or to test and account for multicollinearity (the reader is referred to [42, 46–50]),). The same is observed from studies that used Alternative models such as multivariate linear regressions. For instance, [26] did not test for homoscedasticity, normality, and multi-collinearity in their model. Furthermore, A key issue with the use of the multivariate linear regressions demand model is that the use of levels does raise concerns over stationarity. The use of log levels in the model does not account for potential nonstationary in the variables, nor does it examine integrating relationships between the variables concerned.

Lastly, it is noted that some of the review studies either use time series or cross-sectional data with a relatively small-time span or with a few variables. This was most noticeable in the study by [50] where only 3 variables were used. Some studies had few variables because of the use of the Variance inflation factor (VIF) to remove insignificant variables or variables that are highly correlated with other independent variables. This has the potential problem of shifting the model that is being tested, meaning that the theory being tested by the model has changed. For instance, dropping X_j_ from the equation means that the i^th^ regression coefficient no longer represents the relationship between the Y and X_i_ controlling for X_j_ and any other independent variables in the model.

## 3. Data and methodology

### 3.1 Data description

The empirical analysis in this paper uses national quarterly real house price index series from 2000Q1 to 2019Q4. The house price data is sourced from the Federal reserve bank of Dallas. From the year 2000 to the first quarter of 2017, the data is from ABSA Group Limited (formerly Amalgamated Banks of South Africa Limited). In the ABSA index, house prices are based on loans approved by ABSA and represent the total purchase price of a house. From the second quarter of 2017 onwards, the series is based on South Africa’s First National Bank (FNB) transaction price data from homes financed by FNB. Both the ABSA and FNB indexes are reported at a monthly frequency, the Federal reserve bank of Dallas aggregates the data into a quarterly frequency by taking the arithmetic mean of the corresponding months. The index is the smoothed using a Hodrick-Prescott smoothing function with a lambda of 5. The Federal reserve bank of Dallas retains the ABSA series to extend the quarterly series before 2001 and re-base the spliced index to 2005 = 100 and deflates this series using the personal consumption expenditure (PCE) deflator obtained from the South African Reserve Bank.

The period used and the use of national data is subject to data availability. This study uses national data (i.e., assumes a homogenous South African housing market) because of data limitations. The data that is more granular (provincial level and divided into different types of house levels by price) is not available in the public domain and the time period of such data has been discontinued by ABSA. The data that does exist at a provincial level is mostly managed by banks, property valuation firms and private agents.

In line with previous studies (see for instance, [9–12] and housing demand-supply theoretical framework, this study employs the following macroeconomic fundamental variables: gross domestic product, mortgage rate, exchange rate (USDZAR), affordability (Price to income ratio), Household debt to disposable income, Unemployment rate, Share price index (JSE all share index), and Foreign direct investment. All the data were seasonally adjusted and made real using the consumer price index (CPI). The inclusion of the latter variables and exclusion of variables such as population growth is subject to data availability where most of the independent variables such as population growth are only published in values that are yearly and to decompose the data into higher frequency data (quarterly) using decomposition methods such as cubic spline interpolation might reduce the reliability of our results, i.e., introduce a systematic source of serial correlation in regressors. Other data such as rental income was omitted because they had data with a short time span. The reader must note that the data used does not allow for a more granular comparison at an industry level (i.e., looking at low, medium and high-cost housing market). It is acknowledged that it would have added more value to the thesis. Perhaps this will be looked at in future studies.

### 3.2 Methodology

**First stage**: The first stage of the study determines the optimal lag length of the VECM using an information criterion. There are many different information criterions that maybe applied that also produce different results of which lags to choose. This study will employ the most used criterion, the Akaike information criterion (AIC), defined as the equation below [51]:

$$AIC\left(p\right)=ln\left[\frac{SSR\left(p\right)}{T}\right]+\left(p+1\right)\left(\frac{2}{T}\right)$$

where SSR is the sum of the squared residuals, p is the number of parameters estimated and T is the sample size. It must be noted that when choosing the optimal lags using the AIC there is a tradeoff where an increase in the lag length will increase the goodness of fit of the model and a decrease in lag length will make the model simpler [51].

**Second stage**: The second stage of the study examines the time-series properties using the following unit root tests: Augmented Dickey–Fuller [52] and [53] unit root tests to test for stationarity of the data sets. To apply the VECM, the variables need to be non-stationary (contain a unit-root) in levels and become stationary in the first difference to proceed with the cointegration analysis.

[52] -test can be expressed from this equation:

$$\Delta y_{t}=\beta_{0}+a_{t}+\delta y_{t-1}+\gamma_{1}\Delta y_{t-1}+\gamma_{2}\Delta y_{t-2}+\ldots +\gamma_{j}\Delta y_{t-j}+\epsilon_{t}$$

$$H_{o}:\delta =0\;\left(non\text{-}stationary\right)$$

$$H_{o}:\delta <0\;\left(stationary\right)$$

Where *β*_0_ is the constant, *a*_*t*_ is the time trend, *j* is the number of lags, and *γ*_1_, …, *γ*_*j*_ denote the parameters attached to the lags.

[53] -test can be expressed from this equation:

$$y_{t}=\propto +\rho y_{t-1}+\epsilon_{t}$$

$$H_{0}:\rho =1\;\left(non\text{-}stationary\right)$$

$$H_{1}:\rho <1\;\left(stationary\right)$$

**Third stage**: In the third stage, this study will identify the number of cointegrating vectors to be included in the VECM by utilizing the Johansen cointegrating test. The test will compromise of two likelihood ratio tests developed by [54] which are defined as:

$$Trace\;test:\lambda_{trace\left(r^{*}\right)}=-T\sum_{i=\left(r+1\right)}^{K}log\left(1-\hat{\lambda_{i}}\right)$$

$$Maximum\;eigen\;value\;test:\lambda_{max}\left(r^{*}\right)=-Tlog\left(1-{\hat{\lambda }}_{1+r}\right)$$

Where r* is the number of cointegrating vectors being tested for in each test, *T* is the sample size, $\hat{\lambda }$ is the estimated eigenvalue and *K* are the number of variables. The hypotheses for the test are:

*Trace* *test*:

*H*_0_: *r* ≤ r*, there are r* or fewer cointegrating vectors.
*H*_*a*_: r* < *r* ≤ *K*, there are more than r* cointegrating vectors.

*Maximum eigenvalue test*:

*H*_0_: *r* ≤ r*, there are r* or fewer cointegrating vectors.
*H*_*a*_: *r* = *r* + 1, there are r* + 1 cointegrating vectors.

**Fourth stage**: The study then looks at the short-run and long-run causality relations between macroeconomic fundamentals and housing prices using a vector error correction model. This study uses a vector error correction model (VECM) because it is considered ideal for the analysis of short-run dynamics and long-run co-integration relationship amongst variables while accounting for endogeneity and reverse causality [55]. This study will use the VECM of the form:

$$\Delta ln\left(RHPI\right)=\beta_{0}+\overset{n-1}{\underset{i=1}{\sum \;}}\vartheta_{11}\;\Delta ln\left(RHPI_{t-i}\right)+\overset{n-1}{\underset{i=1}{\sum }}\;\vartheta_{12}\;\Delta ln\left(X_{t-i}\right)+\alpha_{0}+\varepsilon_{t}$$ (1)

where *RHPI* denotes real house prices; X is a vector of explanatory variables, *β*_0_, *α*_0_, *ϑ*_11_ *and ϑ*_12_, are the estimated parameters and *εt* is the random error term. Furthermore, all the variables are in logarithm form except for the mortgage rate and foreign direct investment.

**Fifth stage**: Lastly, the study will analyze long-run deviations of house prices from their macroeconomic fundamental variables and generate forecast variance decompositions and impulse response functions to further examine the impact of shocks from the macroeconomic fundamental variables on real house prices.

There are several limitations of the modelling being used such as the difficulty in choosing the optimal lag. There is a trade-off between increased estimation uncertainty and increased model accuracy from adding lags. Therefore, had to depend on the information criterion tests for the appropriate lag to use. Noting that different information criterion can give different results of which lags to choose. The model focuses on the market and does not focus on hedonic pricing variables such as characteristics of both the property itself.

## 4. Data analysis and discussion of findings

### 4.1 Summary statistics

Table 1 above provides the descriptive statistics and the raw data correlation matrix of variables used in this study. The sample has 80 time series observations during the period 2000Q1 to 2019Q4. It is also noted that the mean of most variables except for real house prices, GDP, affordability, and household debt to disposable income is slightly more than the median. Thus, the data from the variables appear to be skewed to the right. In all the variables, there is low dispersion of data in each variable. In other words, the data is not highly spread out from the mean and this is indicated by the small standard deviations of the data set from each variable.

**Table 1:** Summary statistics.

**PANEL A**										
	**RHPI**	**GDP**	**Mortgage rate**	**Exchange rate**	**Affordability**	**Household debt to disposable income**	**Unemployment rate**	**Share prices**	**FDI**	**PPI**
**Mean**	93.02	2666138	12.721	9.469	107.9	74.663	25.290	59.633	57.809	77.462
**Median**	100.386	2720671	10.607	9.004	109.385	76.493	25.100	55.395	33.735	75.685
**Maximum**	123.586	3161917	24.939	17.574	169.552	103.88	29.300	113.381	1919.482	125.342
**Minimum**	49.991	1927597	6.218	6.519	62.324	45.327	21.000	14.171	-644.598	39.208
**Std. Dev**.	19.607	401613.7	5.965	2.106	32.641	17.181	2.011	33.641	286.986	25.432
**Skewness**	-1.009	-0.439	0.613	1.635	0.387	-0.169	0.079	0.157	3.348	0.288
**Kurtosis**	2.887	1.809	2.015	5.973	2.063	2.015	2.383	1.605	23.830	1.887
**CV (%)**	21.078	15.064	46.893	22.246	30.251	23.012	7.954	56.413	496.435	32.831
**Jarque-Bera**	13.63	7.295	8.254	65.083	4.925	3.615	1.354	6.820	1595.769	5.236
**Probability**	0.001	0.026	0.016	0	0.085	0.164	0.508	0.033	0.000	0.073
**Sum**	7441.593	2.13E+08	1017.66	757.5199	8632.034	5973.032	2023.200	4770.623	4624.747	6196.964
**Sum Sq. Dev**.	30370.19	1.27E+13	2811.095	350.5441	84169.11	23320.22	319.632	89403.880	6506497.000	51095.140
**PANEL B**										
	**RHPI**	**GDP**	**Mortgage rate**	**Exchange rate**	**Affordability**	**Household debt to disposable income**	**Unemployment rate**	**Share prices**	**FDI**	**PPI**
**RHPI**	1.000	0.733	-0.685	-0.700	0.020	-0.038	-0.403	0.595	-0.134	0.550
**GDP**	0.733	1.000	-0.942	-0.558	-0.662	-0.657	0.114	0.959	-0.203	0.952
**Mortgage rate**	-0.685	-0.942	1.000	0.641	0.628	0.657	-0.126	-0.882	0.228	-0.882
**Exchange rate**	-0.700	-0.558	0.641	1.000	0.042	0.055	0.283	-0.392	0.093	-0.385
**Affordability**	0.020	-0.662	0.628	0.042	1.000	0.926	-0.639	-0.751	0.156	-0.797
**Household debt to disposable income**	-0.038	-0.657	0.657	0.055	0.926	1.000	-0.704	-0.766	0.219	-0.823
**Unemployment rate**	-0.403	0.114	-0.126	0.283	-0.639	-0.704	1.000	0.262	-0.171	0.347
**Share prices**	0.595	0.959	-0.882	-0.392	-0.751	-0.766	0.262	1.000	-0.232	0.974
**FDI**	-0.134	-0.203	0.228	0.093	0.156	0.219	-0.171	-0.232	1.000	-0.208
**PPI**	0.550	0.952	-0.882	-0.385	-0.797	-0.823	0.347	0.974	-0.208	1.000
**Observations**	80	80	80	80	80	80	80	80	80	80

Abbreviations: RHP: Real House Prices, GDP: Gross Domestic Product

Source: Authors’ own calculation using EViews

As a requirement, normal skewness is associated with a value that is close to 0. The latter is true for most of the variables except real house prices, real exchange rate and foreign direct investment. Real house prices are highly skewed to the left, real exchange rates and FDI are highly skewed to the right. The rest of the variables have a skewness that is approximately symmetric and mirrors that of a normal distribution. Furthermore, all variables except for the real exchange rate and FDI are platykurtic (because kurtosis<3), which implies that the series of the variables will have many values lower than their respective means. When looking at the Jarque Bera statistic, we see that household debt to disposable income, affordability and unemployment rate have probability values that are above the significance level of 0.05. Therefore, they are normally distributed variables unlike the rest of the variables which have probability values that are below the significance level of 0.05.

Panel B illustrates the raw data correlation matrix of the variables. The results suggest that the Gross domestic product, Affordability, share prices and producer price index are positively correlated with real house prices while the rest of the variables are negatively correlated with real house prices. For instance, GDP, is strongly positively correlated with real house prices, generating correlation coefficient of 0.733 while Real exchange rate and mortgage rate are strongly negatively correlated with real house prices, generating correlation coefficients of -0.7 and -0.685 respectively. It is noted that some of the variables are highly correlated with each other and this might lead to multicollinearity problems that will make it difficult to interpret the coefficients in our regressions. However, note that the latter correlation matrix results are for raw data. Diagnostic and stability tests will be presented for our VECM model.

### 4.2 Model diagnostics

#### 4.2.1 Test for stationarity

The Augmented Dickey–Fuller [52] and [53] unit root tests techniques were used to test for the possibility of stationarity of the data at first differences to check if the data used in the study is first order integrated. Before the tests, the variables were transformed into logarithms to obtain stationarity in variance. The results obtained in Table 2 below, show that for both the [52] (ADF) and [53] (PP) unit root tests, this paper cannot reject the null hypothesis of the presence of unit roots (no stationarity) when the variables are in level form, except for GDP and FDI.

**Table 2:** Stationarity test.

Variables	Level Series						Differenced Series					
	ADF			PP			ADF			PP		
	t statistics	P-value		t statistics	P-value		t statistics	P-value		t statistics	P-value	
**RHPI**	0.325	0.777	n0	1.349	0.954	n0	-1.968	0.047	**	-3.140	0.002	***
**GDP**	3.205	1.000	n0	5.076	1.000	n0	-2.496	0.013	**	-3.109	0.002	***
**Mortgage rate**	-2.372	0.018	**	-2.713	0.007	***	-4.836	0.000	***	-4.066	0.000	***
**Exchange rate**	-0.518	0.489	n0	-0.434	0.523	n0	-6.965	0.000	***	-6.965	0.000	***
**Affordability**	-1.109	0.240	n0	-1.349	0.163	n0	-2.162	0.030	**	-2.584	0.010	**
**HDDI**	-1.152	0.225	n0	-1.913	0.054	*	-2.379	0.018	**	-4.271	0.000	***
**Unemployment rate**	0.297	0.769	n0	0.368	0.788	n0	-4.166	0.000	***	-11.297	0.000	***
**Share prices**	2.089	0.991	n0	2.349	0.995	n0	-6.244	0.000	***	-6.290	0.000	***
**FDI**	-3.818	0.000	***	-8.241	0.000	***	-5.874	0.000	***	-36.101	0.000	***
**PPI**	4.287	1.000	n0	6.916	1.000	n0	-1.359	0.160	n0	-3.806	0.000	***

**Abreviations**: RHP: Real House Prices, GDP: Gross Domestic Product, HDDI: Household debt and disposable,

FDI: Foreign direct investment, PPI: Producer price index

**Notes**:

(*) Significant at the 10%;

(**) Significant at the 5%;

(***) Significant at the 1%. And (no) Not Significant,

*[67] one-sided p-values.

When using the PP unit root test, the null hypothesis of the presence of unit roots is rejected when the variables are in first difference form. However, when using the ADF, this study rejects the null hypothesis of no stationarity for all variables except for PPI. Because the PP unit roots test overcomes the problems of autocorrelation and heteroscedasticity, this study proceeds with the results of the PP unit roots test. Since all the variables are *I*(1) (Stationary after differencing) under the PP unit root test, this study will now proceed with the cointegration test.

#### 4.2.2 Lag order selection

This study used the Unrestricted VAR post estimation for the lag order test. It used both the Akaike information criterion (AIC) and Schwartz information criteria (SIC). The maximum lag length of 3 was shown by the AIC with a value of—-33.47663 and SIC displayed a maximum lag length of 2 with a value of -28.13690. This study uses the rule-of-thumb of selecting the criterion with the lowest value, this is because the lower the value, the less the residual correlation, and the more stable and better the model. Thus, a lag length of 3 was specified.

#### 4.2.3 Test for cointegration

This study applies the Johansen test of cointegration to test for the existence of a long run association among the variables used in this study. The results of Johansen tests are reported in Table 3. The null hypothesis of trace statistic is that there are no more than the rank I cointegrating vectors and the null hypothesis of the maximum eigenvalue statistic states that the number of cointegrating vectors is equal to *r*.

**Table 3:** Cointegration test.

No. of CE(s)	Unrestricted Cointegration Rank Test (Trace)				Unrestricted Cointegration Rank Test (Max Eigenvalue)			
	Eigenvalue	Trace Statistic	Critical Value	Prob.**	Eigenvalue	Max-Eigen Statistic	Critical Value	Prob.**
**None** *	0.652	423.351	239.235	0.000	0.652	80.132	64.505	0.001
**At most 1** *	0.623	343.219	197.371	0.000	0.623	74.109	58.434	0.001
**At most 2** *	0.587	269.110	159.530	0.000	0.587	67.299	52.363	0.001
**At most 3** *	0.543	201.811	125.615	0.000	0.543	59.555	46.231	0.001
**At most 4** *	0.449	142.256	95.754	0.000	0.449	45.355	40.078	0.012
**At most 5** *	0.363	96.901	69.819	0.000	0.363	34.244	33.877	0.045
**At most 6** *	0.296	62.657	47.856	0.001	0.296	26.641	27.584	0.066
**At most 7** *	0.212	36.016	29.797	0.008	0.212	18.105	21.132	0.126
**At most 8** *	0.200	17.912	15.495	0.021	0.200	16.946	14.265	0.018
**At most 9**	0.013	0.966	3.841	0.326	0.013	0.966	3.841	0.326

Trace test indicates 9 cointegrating eqn(s) at the 0.05 level

Max-eigenvalue test indicates 6 cointegrating eqn(s) at the 0.05 level

* denotes rejection of the hypothesis at the 0.05 level

** [68] p-values

**Source**: Auth’rs’ own calculation using EViews

We observe that the null is rejected for *r* = 8 at the 5 per cent significance for the trace statistics and *r*-5 for the eigenvalue statistics in favour of at most 9 and 6 respectively. Therefore, the study finds 9 cointegrating equations. The results suggest there is strong evidence to reject the null hypothesis of no cointegration. Therefore, there exists a long run association between the macroeconomic variables under consideration and house prices. The study has applied one restriction because it’s aim focuses on one cointegrating equation that shows the relationship between house prices and macroeconomic variables under consideration. We then proceed to estimate the long run and short run causal effects of the variables using the Vector Error correction model.

### 4.3 Analysis of findings

#### 4.3.1 Short run relationship

Table 4 above shows the dynamics of the short-run causality between macroeconomic fundamentals and housing prices. Initially from Panel A, it is observed that the co-integration equation coefficient is negative and statistically significant, which implies that the house price series converges to a long-run equilibrium. In other words, the previous quarter deviation of house prices from long run equilibrium due to a change in the explanatory variables is corrected at an adjustment speed of 7 per cent. This study also observes that the mortgage rate is the only variable that is not statistically significant at any lag while most variables significantly impact house prices at the first lag order.

**Table 4:** VECM short-run.

**Panel A**					
	**Coefficient**	**Std. Error**	**t-Statistic**	**Prob**.	
**Constant**	-0.001	-0.006	-0.104	0.459	
**ECM**	-0.070	0.030	-2.378	0.010	
**Panel B**					
**Variable**	**Lag 1 Coef**.	**Lag 2 Coef**.			
Real house price index	0.456 (-0.200) **	-0.296 (-0.249)			
Gross domestic product	0.368 (-0.402)	0.530 (-0.386) *			
Mortgage rate	0.001 (-0.004)	-0.005 (-0.004)			
Exchange rate	-0.056 (-0.029) **	-0.054 (-0.035)*			
Affordability	0.033 (-0.242)	0.504 (-0.245) **			
Household debt to disposable income	0.303 (-0.135)**	-0.220 (-0.132) **			
Unemployment rate	-0.163 (-0.108) *	-0.075 (-0.102)			
Share prices	0.159 (-0.103)*	-0.057 (-0.081)			
Foreign direct investment	0.000 (-0.000)***	0.000 (-0.000)			
Producer price index	0.547 (-0.385)*	0.252 (-0.389)			

Notes:

(*) Significant at the 10%;

(**) Significant at the 5%;

(***) Significant at the 1%. and (no) Not Significant

*[67] one-sided p-values.

Abbreviations: ECM: Error Correction Model

HDDI: Household Debt to Disposable Income

**Source**: Auth’rs’ own calculation

For instance, a statistically significant and negative relationship between house prices and the exchange rate is observed, where a 1 unit increase in the exchange rate is associated with a 0.06 per cent decrease in real house prices ceteris paribus. The results correspond to those of [11] who found a negative and statistically significant relationship between the real effective exchange rate and Swedish house prices during the 1986Q1-2016Q4. The model results also shows that a 1 per cent increase in the affordability (proxy to real disposable income) variable is associated with a 0.03 per cent increase in real house prices ceteris paribus. Similar short run directional effect results were obtained by [43], where their vector error correction model revealed a positive effect of real disposable income on real house prices for Japan (0.9515), Italy (1.1754 and 1.6457), and UK (1.6433).

The model results also show the real household debt to disposable income as a key driver of house prices in the short run. More specifically, a 1 per cent increase in real household debt to disposable income is associated with a 0.65 increase in real house prices ceteris paribus. The effect of foreign direct investment and share prices on house prices is in line with the theoretical hypothesis in which the share prices and producer price index have a positive impact on house prices. For instance, a 1 percent increase in share prices and producer price index is associated with a 0.16 and 0.547 increase in house prices respectively, ceteris paribus. The foreign direct investment is found to have a positive but minor effect on house prices in the short run. The gross domestic product is revealed to have a positive impact on house prices on the second lag at the 10 per cent significance level, where a 1 percent increase in the GDP is associated with a 0.53 percent increase in house prices. Lastly, a 0.163 percent decrease in house prices due to a 1 percent increase in the unemployment rate is also observed.

#### 4.3.2 Long run relationship

Table 5 below presents the Vector error correction model estimation results of the long run causality relationship between macroeconomic fundamentals and house prices during the period 2000Q1 to 2019Q4. Initially, it is noted that all the variables are significant at 1, 5, and 10 per cent level. This study also notes that 76.7 per cent of the real house price variation is explained by the variables as depicted by the R^2^.

**Table 5:** VECM long-run.

	Coefficient	Std. Error	t-Statistic	Prob.
Constant	54.850			
Gross domestic product	3.283	-0.925	-3.549	0.000***
Mortgage rate	-0.012	-0.007	1.802	0.038**
Exchange rate	0.546	-0.066	-8.243	0.000***
Affordability	0.578	-0.110	-5.268	0.000***
Household debt to disposable income	0.671	-0.200	-3.346	0.00***
Unemployment rate	0.981	-0.439	-2.233	0.014**
Share prices	-1.776	-0.244	7.278	0.000***
Foreign direct investment	0.000	0.000	2.561	0.006***
Producer price index	3.125	-0.726	-4.306	0.000***
R-squared	0.767			
Adj. R-squared	0.678			
Breusch-Godfrey serial correlation LM test: (after first difference)	Prob. chi-square = 0.7836 >0.05			
Heteroscedasticity test: Breusch-Pagan-Godfrey	Prob. chi-square = 0.1540 >0.05			
Jarque-Bera Joint	0.9382			

Notes:

(*) Significant at the 10%;

(**) Significant at the 5%;

(***) Significant at the 1%. and (no) Not Significant

**Source**: Auth’rs’ own calculation

The results also show that most of the variables have the anticipated causal directional signs, according to economic theory except for the unemployment rate. For instance, a 1 percent increase in the unemployment rate is associated with a 0.98 percent in real house prices. While the latter results deviate from economic theory, one possible explanation is the link between interest rates and unemployment. An increase in unemployment rate will give rise to an expansionary monetary policy that lowers interest rates to promote economic growth and consequently an increase in house prices.

When observing other variables, the model shows GDP as the most important driver of house prices: real house prices fall by 3.28 percent in the long run-in response to a 1 percent increase in real GDP. The outcome of the impact of GDP on house prices is as expected since a strong economic growth is often expected to stimulate demand for houses which in turn causes a surge in house prices. This is also seen from the empirical results of [28, 56, 57], who found the existence of a positive and statistically significant and long run equilibrium relationship between house prices and GDP. However, several other studies (see, among others, [3, 9, 58–61], either find a negative relationship between house prices and GDP, or do not find a statistically significant positive relationship or do not use the real GDP variable. They mostly cite real GDP measurement errors, econometric methodology and time span used in their studies as the reason behind their empirical results.

The producer price index is the second most important determinant affecting real house prices. Specifically, the results suggest that a 1 percent increase in PPI could lead to a 3.13 percent increase in real house prices, ceteris paribus. The impact of PPI on house prices is similar to the estimation result of 0.43 percent impact of construction cost on China house prices in study done by [62]. Estimation of share prices indicate a significant negative impact on house prices with a coefficient of -1.78. These results imply that a 1 percent decrease in share prices leads to a 1.78 percent decrease in real house prices, ceteris paribus. As in the short-run, the empirical results also show that foreign direct investment has a negligible impact on house prices in the long-run. Another possible explanation could be due to the short time span used in our analyses and due to data limitation or measurement errors in the nature and quality of the FDI data.

The mortgage rate shows an inverse impact on real house prices. The results suggest that a 1 per cent increase in the mortgage rate is associated with a 0.01 percent decrease in real house prices, ceteris paribus. The negative directional effect of mortgage rate on house prices complements the results obtained by [3, 9, 11, 34, 63] who all found a strong and negative long-run relationship between real house prices and mortgage rate. For instance, [56]’s empirical results indicated that 1 per cent increase in mortgage rates will decrease Swedish house prices by approximately 8 per cent. The negative impact of mortgage rate on house prices is as expected, as an increase in mortgage rates will discourage the entry of new home buyers into the housing market resulting in a decrease in the number of houses purchased, and with excess supply of houses, there will be a decrease in house prices contracts [64, 65].

Consistent with our theoretical hypothesis, our results reveal a significant positive relationship between affordability and real house prices at the 1 per cent significant level with a coefficient of 0.58 per cent. Indicating that a 1 per cent increase in housing affordability is associated with a 0.58 per cent increase in real house prices ceteris paribus. This is as expected, since an increase in household affordability means an increase in the purchasing power of households that will lead to an increase in the demand and purchase of houses, which then triggers a surge in house prices by house sellers. When including the USZAR real exchange rate in the VECM, the parameter estimate shows a significant positive coefficient of 0.55, suggesting that individuals (mostly foreign investors) are more likely to make property investment decisions during good market conditions and this is in line with the rational expectation theory doctrine. The theory states that an individual’s decision will be based on past trends and available information in the market. Thus, a better real exchange rate performance today suggests a better performance tomorrow and expected capital gains which will sow the seed for housing demand that eventually lead to an increase in house prices.

The impact of the real exchange rate from this study differs from the theoretical point of view of the expected negative impact of real exchange rate on house prices [66]. For instance, [11] found a negative and statistically significant long run relationship between house prices and real exchange rate in Sweden. On the contrary, there are studies such as [57] who diverge from the theoretical point of view and find a positive and statistically significant impact of the real exchange rate on house prices. The reason for the different outcomes might be related to the misalignment (i.e., overevaluation and undervaluation) of the real exchange rates in each respective country under consideration.

Lastly, this study’s econometric estimates revealed a statistically significant positive relationship between household debt to disposable income and real house prices that is significant at the 1 per cent level. This implies that a 1 per cent increase in the household debt to disposable income is associated with a 0.67 per cent decline in real house prices. One possible explanation for our finding is that a decrease in borrowing constraints induces households, especially those entering the housing market to incur more debt to finance their housing purchase or refinance an existing mortgage or change the composition of the debt held because of higher expected future income from owning a house or the expected increase in the value of houses. This creates more demand for housing and a market with a surplus of buyers and fewer sellers that lead to an increase in house prices.

**Fig 1:**
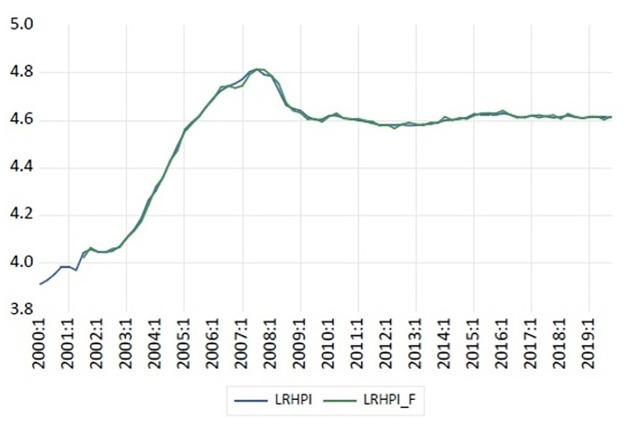
Long-run equilibrium deviation of house prices Time. Source: Auth’rs’ own calculation.

Fig 1 above illustrates the long-run equilibrium deviation of real South African house prices. The LRHPI represents the logged real house price index and LRHPI_F represents the logged forecasted values of fundamental house price index. The deviations, more specifically occurrences of overvaluations between the actual and fundamental house price indices are indicated by the ellipse in the figure. The results from the analysis show that the deviation between fundamental and actual house prices are small in magnitude and negligible except during the 2006Q3-2007Q3. The deviation during the 2006Q3-2007Q3 period does not show any form of persistence or explosive pattern, and there seems to be a relatively quick convergence (closing of the gap) of fundamental house prices and real house prices. It would thus appear that real house price changes at the country-level are explained by changes in long run macro fundamentals during the period under consideration.

#### 4.3.3 Test for sources of volatility

This study will now provide a more depth understanding of the relationships between real house prices and their macroeconomic fundamentals beyond the sample period under consideration. This study will use two widely used techniques that measure sources of volatility, these techniques are Variance decomposition and Impulse response functions.

#### 4.3.4 Variance decomposition

The variance decomposition technique is used to measure the proportion of changes in house prices due to changes in the lagged macroeconomic fundamental variables. Table 6 shows the estimates from the variance decomposition for a 20-quarter time horizon. This study assumes that the period from 1 to 8 represents the short-run period, the rest of the period represents the long-run period.

**Table 6:** Variance decomposition of real house prices.

Period	S.E.	RHP	GDP	M_RATE	ER	AFF	HDDI	UN_RATE	S_PRICES	FDI	PPI
1	0.014	100	0	0	0	0	0	0	0	0	0
2	0.026	92.719	2.685	0.011	0.660	1.175	0.209	0.209	0.115	1.172	1.045
3	0.036	84.124	8.502	0.162	1.222	2.650	0.317	0.109	0.107	0.675	2.131
4	0.049	74.929	13.139	0.123	0.972	3.688	0.564	0.443	0.624	0.464	5.054
5	0.062	69.094	15.099	0.075	0.811	3.421	1.280	0.822	1.018	0.421	7.959
6	0.077	63.813	15.685	0.062	0.700	3.045	2.062	1.399	2.089	0.530	10.617
7	0.093	58.395	16.079	0.110	0.563	2.758	2.992	2.037	3.344	0.797	12.924
8	0.109	53.741	15.854	0.218	0.436	2.405	4.039	2.577	4.625	1.050	15.055
9	0.125	49.622	15.256	0.355	0.339	2.038	5.231	3.051	5.819	1.361	16.926
10	0.141	46.073	14.559	0.520	0.265	1.698	6.360	3.466	6.931	1.650	18.480
11	0.158	43.151	13.836	0.687	0.219	1.406	7.408	3.811	7.919	1.917	19.646
12	0.173	40.679	13.107	0.853	0.197	1.170	8.359	4.088	8.817	2.171	20.558
13	0.189	38.603	12.424	1.008	0.195	0.989	9.235	4.297	9.588	2.389	21.273
14	0.203	36.860	11.794	1.140	0.211	0.856	10.024	4.466	10.260	2.580	21.807
15	0.217	35.396	11.228	1.255	0.239	0.764	10.725	4.600	10.842	2.750	22.202
16	0.230	34.180	10.722	1.352	0.275	0.704	11.345	4.699	11.340	2.892	22.490
17	0.242	33.160	10.272	1.432	0.315	0.670	11.897	4.774	11.769	3.014	22.697
18	0.253	32.305	9.878	1.497	0.358	0.655	12.382	4.830	12.136	3.117	22.842
19	0.264	31.596	9.534	1.550	0.402	0.654	12.808	4.870	12.446	3.203	22.938
20	0.274	31.011	9.233	1.592	0.443	0.662	13.180	4.897	12.710	3.275	22.998
Average	0.147	52.473	11.444	0.700	0.441	1.570	6.521	2.972	6.625	1.771	15.482

**Abbreviations**: S.E. Standard error, RHP: Real House Prices, GDP: Gross Domestic Product, M_RATE: Mortgage rate, ER: Exchange rate, AFF: Affordability, HDDI: Household debt and disposable, UN_RATE: Unemployment rate,

**Source**: Auth’rs’ own calculation

It is observed that over 5 years (20 quarters), on average, a great percentage of forecast error variance in real house prices is explained by the variable itself. While the rest of the variables have a weak influence on real house prices in the short run, however their influence is gradually increasing overtime. For instance, an average of 52.47 percent of the forecast error variance is due to real house prices and the remaining average of 47.53 percent variation is explained by explanatory variables where the PPI accounts for the highest percent among the explanatory variables

PPI approximately accounts for 15.48 percent of the total real house price forecast error variance. The real GDP is shown to be the third largest source with 11.45 percent followed by share prices and household debt to disposable income with a 6.62 percent and 6.52 percent respectively. The affordability is found to have contributed the least source of house price forecast error variance with an average 0.44 percent over the 20 quarters (5 year) time horizon.

#### 4.3.5 Impulse response functions

**Fig 2:**
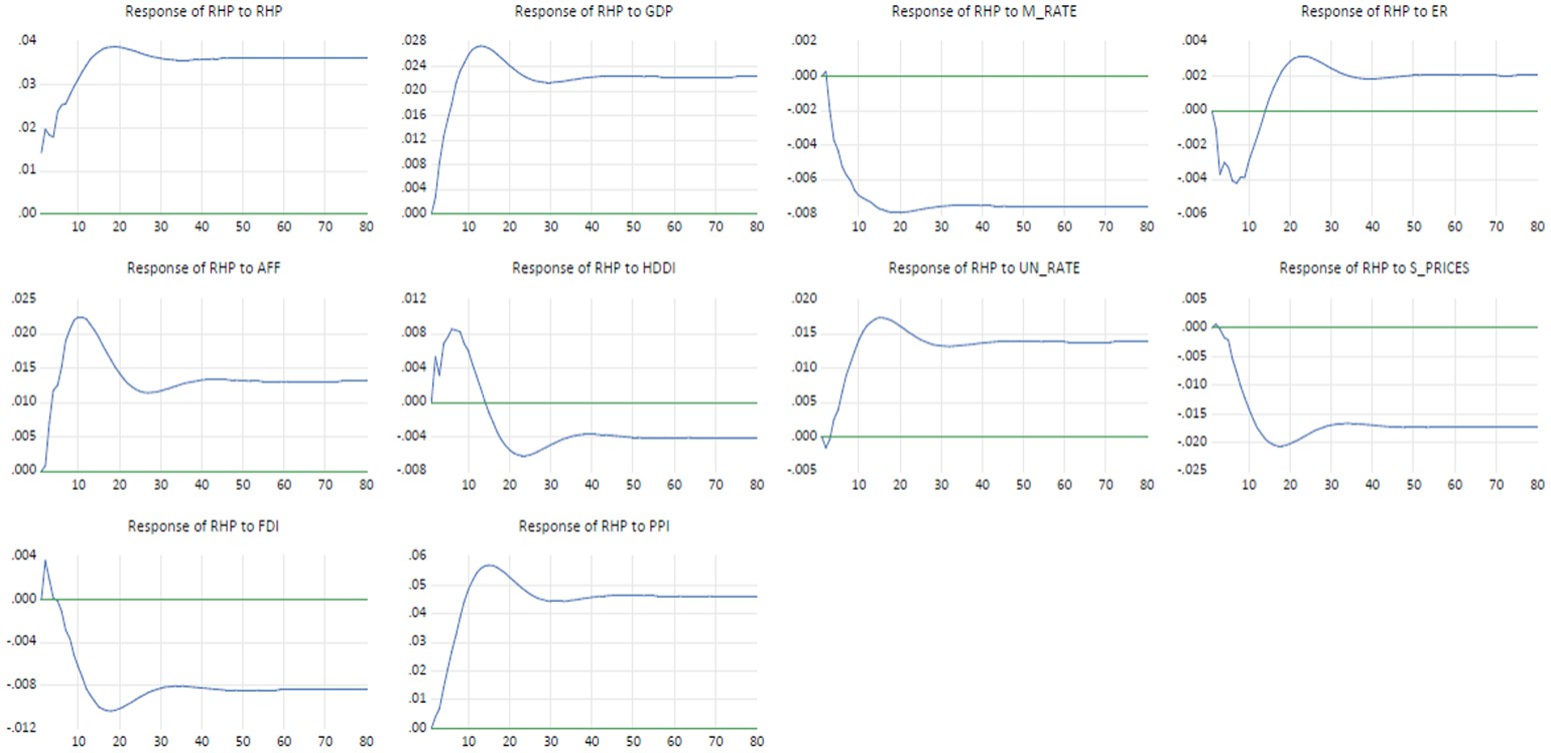
Real house price impulse response. Abbreviations: RHP: Real House Prices, GDP: Gross Domestic Product, M_RATE: Mortgage rate ER: Exchange rate, AFF: Affordability, HDDI: Household debt and disposable, UN_RATE: Unemployment rate, Source: Auth’rs’ own calculation.

Impulse response functions determine the dynamic response of real house prices from a one-unit shock of each explanatory variable over time. Furthermore, they determine whether the shocks have a negative or positive effect and whether they are temporary or have long run effects. Fig 2 below shows the real house price impulse responses associated with a one-standard-deviation positive shock from each of the explanatory variables of the VECM over a period of 80 quarters.

Initially, this study observes that an increase in house prices in response to a one standard deviation disturbance in current house prices, future house prices initially increase by approximately 0.04 percent and reach its peak at the 18^th^ quarter, and then gradually decreases reaching a new equilibrium level that is above the initial level. This implies that the current house prices change has a greater influence on people’s expectation of next quarter’s price and over the long-time horizon.

In response to a GDP shock, initially, real house prices significantly increase by patterns approximately 0.028 and start to fall towards a new equilibrium at the 12^th^ quarter. Similar impulse response movements are observed for affordability, PPI and unemployment rate variables. The impulse response from the unemployment rate was unexpected as was the case in the VECM long run model results. In theory, an increase in the unemployment rate leads to fewer individuals who can afford houses, eventually drives down house prices

A one standard deviation disturbance originating from mortgage rate is associated with significantly negative and persistent decrease in house prices of approximately 3 per cent until it reaches its new low equilibrium level at the 18^th^ quarter. The results are as expected, since a rise in the mortgage rate means an increase in the cost of financing a house which leads to a decrease in the demand for housing, resulting in a decrease in house prices.

Similar impulse responses patterns to those of a mortgage rate shock are also observed from FDI and share price shocks. As in the long-run VECM, the impulse response of house prices results from FDI and share prices are not in line with theory where we expect an appreciation of house prices from an increase in share prices and FDI.

The impact of a one standard deviation disturbance of the real exchange rate on real house prices initially causes house prices to gradually decrease in the first 10 quarters and then increases to reach a new and higher steady state at the 40^th^ quarter. This is as expected, the initial decrease in house prices could have been from a decrease in the demand of house from foreign investors due to a stronger rand and the appreciation of house prices could have stem from the increase in purchasing power of the local investors.

Lastly, this study observes an initial sharp increase in house prices of approximately 0.8 per cent, that peaks at the 9^th^ quarter due to a one standard deviation shock from household debt to disposable income. The house prices then decrease by 0.12 percent to reach a new equilibrium level that is below the initial level. The initial increase could be explained by an increase in purchasing power of house buyers due to their access to funding in the form of debt and the decrease in later quarters that leads to a low steady state level could have been caused buy house buyers who were unable to service their debt, leading to some losing their houses. With increased supply of houses this leads to a decrease in house prices.

#### 4.3.6 Robustness of the model

Several model stability and diagnostic tests were performed (the reader is referred to Table A1 in S1 Appendix) on the VECM, the tests included the Jarque-Bera test for normality, Breusch-Godfrey serial correlation LM test and the Breusch-Pagan-Godfrey heteroskedasticity test. The tests show no significant autocorrelation or heteroscedasticity that is present in the model and further showed that the VECM mirrors a normal distribution. This study then proceeded to test for the stability of the parameter estimates by performing some robustness check of the long run causality test between house prices and macro fundamental variables by using fully modified ordinary least squares regression (FMOLS). The results are presented below in Table 7. From the outset, it is noted that the variables from FMOLS regression have the same directional causal signs as that of the VECM except for the exchange rate, share prices and household debt to disposable income.

**Table 7:** Fully modified least squares.

	Coefficient	Std. Error	t-Statistic	Prob.
Gross domestic product	0.946	0.215	4.401	0.000***
Mortgage rate	-0.005	0.001	-4.143	0.000***
Exchange rate	-0.005	0.018	-0.260	0.796
Affordability	0.936	0.020	47.554	0.000***
Household debt to disposable income	-0.135	0.037	-3.637	0.001***
Unemployment rate	0.314	0.101	3.117	0.003***
Share prices	0.120	0.046	2.592	0.012**
Foreign direct investment	0.000	0.000	-1.870	0.066*
Producer price index	1.067	0.181	5.883	0.000***
Constant	-15.792	2.792	-5.656	0.000***

**Source**: Authors’ own calculation

The difference in the directional causal signs might be due to the short time span used in our analyses due to data limitation or measurement errors in the nature and quality of data used. However, the directional signs can also be theoretically justified. For instance, the negative impact of household debt to disposable income on house prices might be explained by an increase in disposable income which leads to more individuals qualifying for mortgages to buy house which will consequently lead to more demand and a rise in house prices. The 0.12 percent increase in house prices from a 1 percent increase in share prices might originate from foreign investors who were attracted by the increase in South African share prices where some of their investments involves investing in the property markets that leads to a surge in house prices. Lastly the exchange rate is observed to have an insignificant impact on house prices at all levels of significance.

### Implications of the study

The results generated in this study have important implications as they provide greater insights into some of the macroeconomic drivers behind South African house prices and their direction of travel from macroeconomic shocks. This is important in terms of financial stability, for instance, the identification of the mortgage rate as a least insignificant driver of South African house prices may shed more light into the mortgage loan over indebtedness by many South African and their associated potential high risk that may undermine financial stability in the absence of macroprudential intervention. From the study, it is also possible to draw conclusion on the sustainability of house prices levels in South Africa where the continued house price appreciation as from the study may cause for concern of a house price bubble formation in the near future. Therefore, several policies may be pursued by the policymakers to minimize the potential bubble risks.

## 5. Conclusion and recommendations

This study examined the short and long term co-integrating relationship between house prices and selected macroeconomic fundamental factors using the Vector Error Correction Model during the 2000Q1-2019Q4 period. The selected macroeconomic fundamentals were: Gross domestic product, mortgage rate, exchange rate (USDZAR), affordability (price to income ratio), household debt to disposable income, unemployment rate, share prices, foreign direct investment, and producer price index.

Several key findings have been identified in the study. The results suggest that most of the macro-fundamental variables have a significant impact on South African house prices at the first lag order in the short-run at the 1 per cent significance level except for the mortgage rate which was not statistically significant at any significance level. In addition, the results suggest that the house prices series converges in the long-run with an adjustment factor of 7 per cent. We also find existence of a significant long-run relationship between South African real house prices and the selected macroeconomic fundamentals. However, GDP and PPI seem to have the greatest impact over the period under review. Similar observations were made by [3, 22, 26] who found that GDP has a positive and significant impact on house prices.

Variance decomposition suggest that the greatest source of variation in future real house prices, in current real house prices. In addition, the impulse response functions suggest that a one standard deviation shock from the GDP, PPI, exchange rate, affordability, and unemployment rate exerts a positive and persistent effect on house prices. Real house prices maintained a higher level than the initial equilibrium level. While the rest of the variables had a negative and persistent shock that left real house prices at a much lower equilibrium level than the initial positive equilibrium.

The above observations would call for a more balanced and complementary combination of key fiscal and monetary policies to ensure growth in the house market. For example, an expansionary monetary policy may accelerate house price growth through low interest rates that give rise to high demand for mortgage loans and houses. A complementary fiscal policy would be an expansionary policy where the government issues bonds that may “crowd-out” some of private investments leading to a rise in interest rates which may negatively affect house price growth.

Despite South Africa’s well-developed credit markets, evidence suggests that access to housing finance is mostly restricted to high income earners [20]. Private-public partnership might be one of the key interventions that may be applied to accelerate this and thus close the gap between low-income earners, by providing low household income earners with houses at subsidized prices and/or reasonable price spread policies. To balance the excess demand of houses from the latter mentioned policy, an introduction of tax incentives for construction firms may increase productivity of affordable houses and an increase of competition in the housing market opening it up to smaller construction companies as well. The objective of these interventions would be to balance demand and supply of houses and thus stabilise house prices.

## Supporting information

S1 Appendix (DOCX)
