# Supplementary material for: The dynamic effect of macroeconomic factors on housing prices: Evidence from South Africa
Source: PLoS One. 2023 Nov 29;18(11):e0290552. doi: 10.1371/journal.pone.0290552 (PMC10686468; doi:10.1371/journal.pone.0290552)
Supplement: S1 Appendix — (DOCX) [file pone.0290552.s001.docx]

**Appendix**

| **Table A1** |  |
| --- | --- |
| **Variable name** | **Data Source** |
| Real House price index | Federal Reserve bank of Dallas |
| Gross domestic product | International monetary fund |
| Mortgage rate | International monetary fund |
| Exchange rate | International monetary fund |
| Affordability (Price to income ratio) | Organisation for Economic Co-operation and Development |
| Household debt to disposable income | South African Reserve Bank |
| Unemployment rate | South African Reserve Bank |
| Share prices | Organisation for Economic Co-operation and Development |
| Foreign direct investment | South African Reserve Bank |
| Producer price index | Organisation for Economic Co-operation and Development |
| Consumer price index | Federal Reserve bank of Dallas |
|  | |

| **Table A2** |  |
| --- | --- |
| **Variable name** | **Definition (Estimation)** |
| Gross domestic product | This is expenditure-based GDP expressed in billions of rands. It is the total final expenditures at purchasers’ prices (including the free on-board value of exports of goods and services) less free on-board value of imports of goods and services. |
| Mortgage rate | Basic or default interest rate that you could be charged when borrowing for big purchases such as a home, it is linked to the repo rate, when the repo rate goes up or down, the mortgage interest rate changes by the same margin. |
| Exchange rate | It is calculated as the price of South Africa’s currency (Rand) per US dollar. |
| Affordability (Price to income ratio) | The price to income ratio is the nominal house price index divided by the nominal disposable income per head. |
| Household debt to disposable income | It is calculated as household debt as a percentage of household gross income. |
| Unemployment rate | Calculated as the proportion of the labour force (Employed + Unemployed) that is unemployed according to the formal definition of unemployed persons from Statistics South Africa |
| Share prices | Share price indices are calculated from the prices of common shares of companies traded on national or foreign stock exchanges. They are determined by the stock exchange, using the closing daily values for the monthly data, and expressed as simple arithmetic averages of the daily data. |
| Foreign direct investment | Are net inflows of investment to acquire a lasting management interest (10 percent or more of voting stock) in an enterprise operating in an economy other than that of the investor. It is the sum of equity capital, reinvestment of earnings, other long-term capital, and short-term capital as shown in the balance of payments. This series shows net inflows (new investment inflows less disinvestment) in the reporting economy from foreign investors and is divided by GDP. |
| Producer price index | Producer price indices in manufacturing measure the rate of change in prices of products sold as they leave the producer. They exclude any taxes, transport, and trade margins that the purchaser may have to pay. |
| Consumer price index | Headline consumer price index - measured in terms of the annual growth rate and in index, 2015 base year with a breakdown for food, energy and total excluding food and energy. |
|  |  |

| **Table A3: Diagnostic and Stability Tests** | | | |  |  |  |  |
| --- | --- | --- | --- | --- | --- | --- | --- |
| **VEC Residual Serial Correlation LM Tests** | | | |  |  |  |  |
| Date: 09/16/21 Time: 04:18 | | |  |  |  |  |  |
| Sample: 2000Q1 2019Q4 | |  |  |  |  |  |  |
| Included observations: 77 | |  |  |  |  |  |  |
|  |  |  |  |  |  |  |  |
| Null hypothesis: No serial correlation at lag h | | | |  |  |  |  |
|  |  |  |  |  |  |  |  |
| Lag | LRE* stat | df | Prob. | Rao F-stat | df | Prob. |  |
|  |  |  |  |  |  |  |  |
| 1 | 108,93 | 100 | 0,2545 | 1,09895 | (100, 269.6) | 0,2748 |  |
| 2 | 84,504 | 100 | 0,8666 | 0,81888 | (100, 269.6) | 0,8771 |  |
| 3 | 89,3808 | 100 | 0,7678 | 0,87309 | (100, 269.6) | 0,7836 |  |
| **Source: Authors' own calculation using EViews** | | | | |  |  |  |

| **Table A4** |  |  |  |  |  |  |  |
| --- | --- | --- | --- | --- | --- | --- | --- |
| **VEC Residual Heteroskedasticity Tests (Levels and Squares)** | | | | | |  |  |
| Date: 09/16/21 Time: 04:19 | | |  |  |  |  |  |
| Sample: 2000Q1 2019Q4 | |  |  |  |  |  |  |
| Included observations: 77 | |  |  |  |  |  |  |
|  |  |  |  |  |  |  |  |
| Joint test: |  |  |  |  |  |  |  |
| Chi-sq | df | Prob. |  |  |  |  |  |
| 2379,314 | 2310 | 0,154 |  |  |  |  |  |
|  |  |  |  |  |  |  |  |
| **Source: Authors' own calculation using EViews** | | | | |  |  |  |

| **Table A5** |  |  |  |  |  |  |  |
| --- | --- | --- | --- | --- | --- | --- | --- |
| **Normality Test** |  |  |  |  |  |  |  |
| **Component** | **Jarque-Bera** | **df** | **Prob.** |  |  |  |  |
| 1 | 11,8453 | 2 | 0,0027 |  |  |  |  |
| 2 | 9,19855 | 2 | 0,0101 |  |  |  |  |
| 3 | 9,00225 | 2 | 0,0111 |  |  |  |  |
| 4 | 0,80927 | 2 | 0,6672 |  |  |  |  |
| 5 | 0,15715 | 2 | 0,9244 |  |  |  |  |
| 6 | 1,54743 | 2 | 0,4613 |  |  |  |  |
| 7 | 4,7848 | 2 | 0,0914 |  |  |  |  |
| 8 | 2,78225 | 2 | 0,2488 |  |  |  |  |
| 9 | 1,28081 | 2 | 0,5271 |  |  |  |  |
| 10 | 0,1519 | 2 | 0,9269 |  |  |  |  |
|  |  |  |  |  |  |  |  |
| Joint | 869,346 | 935 | 0,9382 |  |  |  |  |
|  |  |  |  |  |  |  |  |
| **Approximate p-values do not account for coefficient estimation** | | | | | |  |  |
| **Source: Authors' own calculation using EViews** | | | | |  |  |  |
